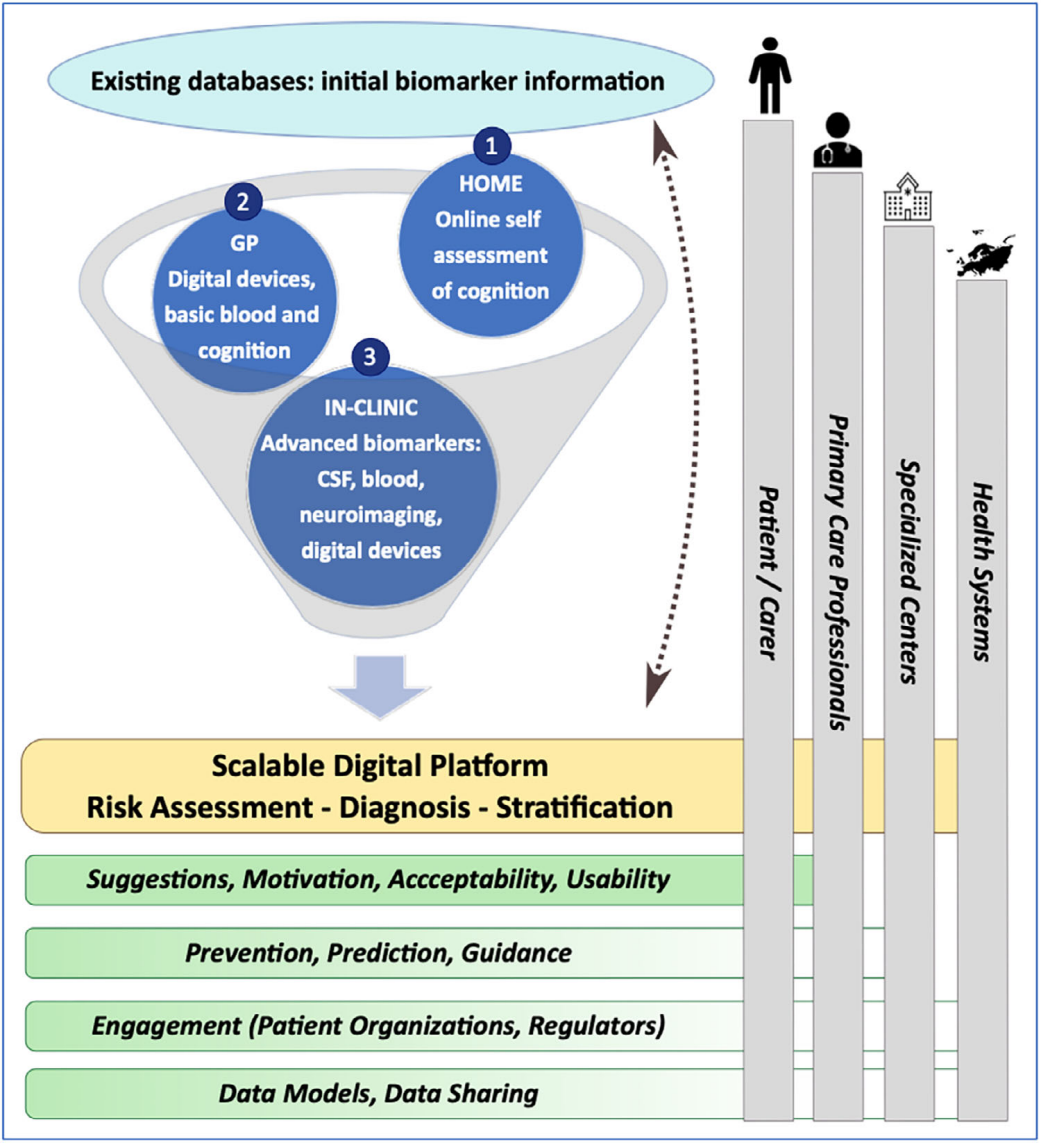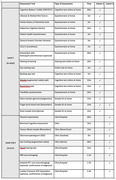# Screening for Alzheimer’s disease in primary care using an AI driven screening platform: design of the PREDICTOM study

**DOI:** 10.1002/alz.087279

**Published:** 2025-01-09

**Authors:** Anna‐Katharine Brem, Zunera Khan, Mark Ashworth, Nicholas J. Ashton, Sigurd Brandt, Anne Corbett, Ana Diaz, Holger Fröhlich, Martha Therese Gjestsen, Dianne Gove, Sandeep Kaushik, Gaby Marquardt, Matthias Müllenborn, Spiros Nikolopoulos, Lucas Paletta, Audun Osland Vik‐Mo, Robert Perneczky, Silvia Russegger, Timo Schirmer, Amied Shadmaan, Ana Bea Solana Sanchez, Dag Aarsland, PREDICTOM Consortium

**Affiliations:** ^1^ King’s College London, London United Kingdom; ^2^ University Hospital of Old Age Psychiatry, University of Bern, Bern Switzerland; ^3^ Department of Old Age Psychiatry, King's College London, London, london United Kingdom; ^4^ King's College London, London United Kingdom; ^5^ Institute of Neuroscience and Physiology, University of Gothenburg, Mölndal Sweden; ^6^ King’s College London, Institute of Psychiatry, Psychology & Neuroscience, Maurice Wohl Clinical Neuroscience Institute, London United Kingdom; ^7^ GN brainworks, Ballerup Denmark; ^8^ College of Medicine and Health, University of Exeter, Exeter United Kingdom; ^9^ Alzheimer Europe, Luxembourg Luxembourg; ^10^ Fraunhofer Institute for Algorithms and Scientific Computing SCAI, Sankt Augustin Germany; ^11^ Centre for Age‐Related Medicine – SESAM, Stavanger University Hospital, Stavanger Norway; ^12^ GE Healthcare, Munich Germany; ^13^ Siemens Healthineers, Erlangen Germany; ^14^ Novo Nordisk, Copenhagen Denmark; ^15^ Centre for Research & Technology Hellas, Thessaloniki Greece; ^16^ JOANNEUM RESEARCH Forschungsgesellschaft mbH, Graz, Styria Austria; ^17^ Stavanger University Hospital, Stavanger Norway; ^18^ LMU University Hospital, Munich Germany; ^19^ Department of Neuroradiology, University Hospital LMU, Munich Germany; ^20^ GE Healthcare, London United Kingdom; ^21^ King's College London, London, England United Kingdom

## Abstract

**Background:**

Recent developments in physiological and digital biomarkers provide an opportunity to shift the first diagnostic steps to the home‐setting, thus allowing earlier detection and treatment of Alzheimer’s disease (AD). Blood‐based, magnetic resonance imaging, electrophysiological, digital and microbiome biomarkers have shown great promise and call for an evaluation of their accuracy, feasibility and safety in primary care and the community. The aim of PREDICTOM is to develop and test the accuracy of an artificial intelligence (AI) driven screening platform for the prediction and early detection of AD and to extend the clinical pathway to home‐based screening using established and novel biomarkers.

**Method:**

PREDICTOM is a multicentre (Norway, UK, Belgium, France, Switzerland, Germany, Spain) cohort study (Figure 1) using a cloud‐based platform that stores a digitalised journey for each participant and provides a collection of AI algorithms and tools for risk assessment, early diagnosis and prognosis. We will use data from existing cohorts to guide the analytic strategy of the study, which consists of two consecutive cohort phases: 1) Home‐based assessment of individuals over the age of 50y with increased risk of developing AD (N=4000); 2) In‐clinic assessment of participants selected from cohort 1 based on biomarker results indicating high (N=415) or low (N=200) risk of AD. Cohort 1 (Level 1, Table 1) includes at‐home screening collecting digital (cognition, hearing, eye‐tracking, questionnaires) and physiological (finger‐prick blood, saliva, stool) data for biomarker analysis. Cohort 2 (Table 1) continues with a more complex biomarker collection including EEG, MRI, blood, cognition, hearing, and eye‐tracking (Level 2) followed by a diagnostic evaluation (Level 3) to confirm or rule out AD using established biomarkers (cerebrospinal fluid, plasma, or amyloid PET). Participants included within the first two years will complete a 12‐month follow‐up visit. We involve the public and will develop a system feedback concept for participants and primary care providers.

**Result:**

First results are expected to be disseminated in 2025.

**Conclusion:**

PREDICTOM will provide an important stepping stone towards the use of AI‐driven algorithms for the early detection of AD using existing and novel biomarkers and their integration into a well‐defined and comprehensive clinical pathway.

This Project Is Supported By The Innovative Health Initiative Joint Under taking (IHIJU) Under Grant Agreement No 101132356. The JU Receives Support From The European Union’s Horizon Europe Research AndInnovation Programme. This Work Was Funded By UK Research And Innovation (UKRI) Under The UK Government’s Horizon Europe Funding Guarantee [UKRI Reference Number: 10083181]. In Switzerland The University Of GenevaIs Funded For PREDICTOM By The Swiss State Secretariat For Education Research And Innovation (SERI‐Ref‐1131 52304).